# Amperometric catechol biosensor based on laccase immobilized on nitrogen-doped ordered mesoporous carbon (N-OMC)/PVA matrix

**DOI:** 10.1088/1468-6996/15/3/035005

**Published:** 2014-06-10

**Authors:** Meiqing Guo, Hefeng Wang, Di Huang, Zhijun Han, Qiang Li, Xiaojun Wang, Jing Chen

**Affiliations:** 1 College of Mechanics, Taiyuan University of Technology , Taiyuan 030024, People’s Republic of China; 2 Institute of Applied Mechanics and Biomedical Engineering, Taiyuan University of Technology ,Taiyuan 030024, People’s Republic of China; 3 Shanxi Key Laboratory of Material Strength and Structural Impact, Taiyuan University of Technology , Taiyuan 030024, People’s Republic of China; 4 Shanxi Coal Bed Methane Co., Ltd , Taiyuan 030024, People’s Republic of China; 5 Authors to whom any correspondence should be addressed

**Keywords:** biosensor, laccase, N-OMC, PVA, electrical properties

## Abstract

A functionalized nitrogen-containing ordered mesoporous carbon (N-OMC), which shows good electrical properties, was synthesized by the carbonization of polyaniline inside a SBA-15 mesoporous silica template. Based on this, through entrapping laccase onto the N-OMC/polyvinyl alcohol (PVA) film a facilely fabricated amperometric biosensor was developed. Laccase from *Trametes versicolor* was assembled on a composite film of a N-OMC/PVA modified Au electrode and the electrochemical behavior was investigated. The results indicated that the N-OMC modified electrode exhibits electrical properties towards catechol. The optimum experimental conditions of a biosensor for the detection of catechol were studied in detail. Under the optimal conditions, the sensitivity of the biosensor was 0.29 A*M^−1^ with a detection limit of 0.31 *μ*M and a linear detection range from 0.39 *μ*M to 8.98 *μ*M for catechol. The calibration curve followed the Michaelis–Menten kinetics and the apparent Michaelis–Menten 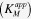 was 6.28 *μ*M. This work demonstrated that the N-OMC/PVA composite provides a suitable support for laccase immobilization and the construction of a biosensor.

## Introduction

Phenols are byproducts of the large-scale production of drugs, dyes, antioxidants, paper pulp and pesticides, and cause ecologically undesirable effects [1, 2]. Most phenols exhibit different toxicities and their determinations are very important for evaluating the total toxicity of an environmental sample. Various methods like spectrophotometry and high performance liquid chromatography have been proposed for the determination of phenolic compounds. However, phenolic compounds are subjected to chromatographic separation before detection in general and the separation takes time and often requires pre-concentration. Many biosensors have been developed in the past using the catalytic activity of the redox enzymes such as tyrosinase, peroxidase, laccase, etc for the determination of phenols [3–6]. Laccase (Lac) are phenoloxidases and also the simplest members of the multi-copper family. Moreover, laccase have the capability to catalyze the 4-electron reduction of oxygen to water with concomitant oxidation of a broad range of substrates such as phenols, anilines, benzenethiols, and phenothiazines [7]. Based on this, laccase can be used in the development of biosensors for the determination of phenolic compounds in aqueous solutions. However, most applications require laccase immobilization, which plays a crucial role for the performance of laccase biosensors. So far, many methods and materials of laccase immobilization have been reported, such as carbon-fiber, a carbon nanotubes-chitosan composite and a sol-gel matrix of diglycerysilane [8–10].

In recent reports [11–13], mesoporous materials have attracted growing interest because of their potential applications as optical, electronic, and electrochemical devices. Ordered mesoporous carbon (OMC) have been used in the immobilization of larger biomolecule matrices due to their large pore size, uniform and tailored pore structure, huge surface areas, high loading capacity, good biocompatibility and electrical conductivity [14–18]. In addition, biomolecules generally display good stability due to some folding forces of pores in the pores of mesoporous materials, and can be firmly incorporated into the matrix without the aid of other cross-linking reagents [19, 20]. However, the poor film-forming property and conductivity of OMC cannot well satisfy its application as enzyme supports of biosensors.

According to previous papers, the incorporation of foreign atoms into OMC can enhance the optical, electrical, semiconducting and surface properties, etc [21–24]. We reported the synthesis of the copper-containing ordered mesoporous carbon and found that the electrochemical properties were improved [21]. In addition, Villalpando-Paez *et al* reported on the synthesis of N-doped single-walled carbon nanotubes and observed that the relative electrical conductivity of the strands increased with increasing nitrogen concentration [22]. By virtue of the OMC’s particular properties, doping OMC with heteroatoms has received vital concern and even promised access to a wide range of applications. However, the application of OMC doping heteroatoms into the enzyme biosensor was rarely reported.

Accordingly, in the present study, a facile method was firstly used to incorporate nitrogen in the OMC frameworks (N-OMC), and the laccase biosensor was fabricated by immobilizing laccase in the N-OMC/PVA composite film. The morphology and microstructure were then investigated by field-emission transmission electron microscopy (FE-TEM), x-ray diffraction (XRD), x-ray photoelectron spectroscopy (XPS), nitrogen adsorption and energy dispersive x-ray spectroscopy (EDX). Cyclic voltammetry (CV), linear range, detection limit, current sensitivity and the Michaelis constant were used to characterize the proposed laccase biosensor. The N-OMC/PVA/Lac film modified Au electrode was expected to improve some disadvantages of the amperometric laccase biosensor.

## Materials and methods

### Materials

Mesoporous molecular sieves SBA-15 (pore size: 6.0–7.0 nm, specific surface area: 900–1200 m^2^ g^−1^, pore volume: 1.26 cm^3^ g^−1^) was acquired from Fudan University (Shanghai, China). Laccase from *Trametes versicolor* (EC 1.10.3.2, ≽ 10 unit mg^−1^) was purchased from Sigma Co. PVA with 1750 ± 50 degree of polymerization and 98% of degree of hydrolysis was supplied by the Experimental Chemical Plant of Tianjin University (Tianjin, China). All chemicals were of analytical grade. 0.1 M phosphate buffer solution (PBS) consisting of NaH_2_PO_4_ and Na_2_HPO_4_ was employed as the supporting electrolyte. The pH value of the desired solution was adjusted with 0.1 M NaOH or 0.1 M H_3_PO_4_ solution. All aqueous solutions were prepared with bi-distilled water.

### Apparatus

All the electrochemical measurements were performed on a PARSTAT 2263 electrochemical workstation (Princeton, USA). The electrochemical measurements were carried out with a conventional three-electrode system. N-OMC/PVA/Lac or OMC/PVA/Lac modified Au electrodes (4 mm diameter) were used as working electrodes with a saturated calomel electrode (SCE) as a reference electrode and a Pt wire (1 mm diameter) as an auxiliary electrode in all cases. Nitrogen adsorption-desorption isotherm was measured at 77 K on a NOVA2000 Autosorb Sorption Analyzer (Quantachrome Corporation, USA). The specific surface area was calculated with the Brunnauer–Emmett–Teller (BET) method. The pore size and pore volume were acquired from the adsorption branch of the isotherms by the Barrett–Joyner–Halenda (BJH) method. The morphology and composition of N-OMC was characterized with transmission electron microscopy (TEM, Tecnai-G2F20, Philips), EDX analysis with Vario EL (Elementar, Germany) and x-ray photoelectron spectroscopy (PHL1600ESCA, XPS). The powder XRD patterns were collected on a Rigaku D/max 2500 V pc^−1^ diffractometer (Rigaku Corporation, Japan) using Cu K*α* (*λ* = 0.154 nm) radiation.

Electrochemical impedance spectroscopy (EIS) was measured in 5.0 mM K_3_[Fe(CN)_6_]/K_4_[Fe(CN)_6_] (1:1) solution supported by 0.1 M KCl in the frequency range from 10^−2^ Hz to 10^5^ Hz. (Number of point per frequency decade was 30 and AC was 10 mV rms^−1^). Electrochemical catalytic behaviors of the electrodes towards catechol oxidation were characterized by cyclic voltammetry at a scan rate of 50 mV*s^−1^ with the applied potential range from −0.2 V to 0.8 V (versus SCE). All measurements were conducted at room temperature.

### Preparation of N-OMC

The nitrogen-containing ordered mesoporous carbon, denoted hereafter as N-OMC, was synthesized as follows. 0.4 g SBA-15 and 3.0 g purified aniline were added homogeneously to 500 ml of 1 M HCl aqueous solution consisting of 3.2 g ammonium persulfate (APDS) under stirring for 12 h. The amount of aniline used was expected to fill up the pores of SBA-15, and the polymerization can be carried out in the pores of SBA-15. The resulting sludge was dried at 100 °C for 12 h. Afterwards, the obtained mixture was again impregnated with 3.2 g of APDS dissolved by 100 mL of 1 M HCl aqueous solution, and again dried at 60 °C for 36 h. The material thus obtained was heated to 950 °C at 5 °C min^−1^ in argon atmosphere and kept for 6 h. Finally, the silica framework was removed by putting the resultant mixture obtained in 25 wt% hydrofluoric acid for 12 h. N-OMC was obtained by pump filtration, washing several times with ethanol and doubly distilled water, respectively, and being dried at 120 °C in an oven. The pure OMC was also synthesized using glucose as a carbon resource. The detailed procedure was described in our previous literatures [21].

### Immobilization of laccase

Before immobilization, N-OMC was modified by means of oxidation treatments in 98 wt% H_2_SO_4_, and followed by 68 wt% HNO_3_ to improve its solubility. The immobilization process was performed by the following approach: 4 mg acidified N-OMC were dispersed into 4 mL 1 mg ml^−1^ laccase (in pH 5.0 PBS) and then the suspension was kept at 4 °C for 24 h under stirring and was separated by centrifugation under the conditions of 8000 r min^−1^ in rotating speed and 15 min in resting time. Finally, the N-OMC/Lac was mixed with 4 ml of 0.1% PVA solution, which was denoted as N-OMC/Lac/PVA.

### Electrode modification

The Au electrode (4 mm diameter) was used as the substrate electrode. Before experiment, it was polished with 1.0, 0.3 and 0.05 *μ*m alumina slurry and rinsed thoroughly with double distilled water, and then sonicated in double distilled water and allowed to dry at room temperature. Then, 10 *μ*l N-OMC/Lac/PVA solution was dropped on the surface of the electrodes and allowed to dry under ambient conditions for 2 h at 4 °C. The thickness of the N-OMC/Lac/PVA film was controlled by the volume of N-OMC/Lac/PVA solution used. At last, the modified electrode was rinsed with doubly distilled water twice, and then the N-OMC/Lac/PVA modified electrode was obtained. The OMC/Lac/PVA modified electrode was also prepared by the above-described method. When not in use, the electrodes were stored at 4 °C.

## Results and discussion

### Characterization of N-OMC

**Figure 1:**
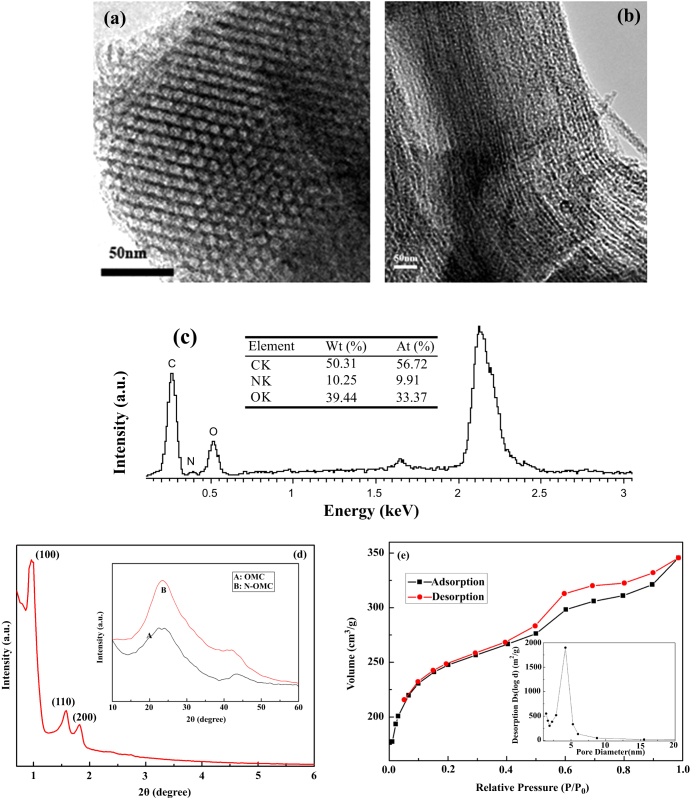
TEM images of N-OMC taken (a) along and (b) perpendicular to the channel direction, (c) EDX graph of N-OMC, (d) XRD spectrum of N-OMC, and (e) nitrogen adsorption–desorption isotherm of N-OMC. Inset of figure 1(d): wide angle XRD patterns of OMC (A) and N-OMC (B).

Figures 1(a), (b) shows TEM images of the synthesized N-OMC viewed along and perpendicular to the direction of the ordered pore arrangement. It was observed that the synthesized N-OMC possesses an ordered surface structure with a pore size of 4.2 nm. The carbon nanorods were interconnected by spacers, which were constituted by the carbon filled in the channel-interconnecting micropores within the SBA-15 wall. The EDX spectrum in figure 1(c) clearly shows the presence of an N element and the absence of an Si element, which clearly demonstrates that the SBA-15 template was completely removed, and the N element was incorporated into the carbon framework. In addition, from the EDX spectrum, the atom percentage of doped nitrogen within N-OMC can be estimated to be 9.91%.

The ordered arrangement of the N-OMC materials gives rise to the well-resolved XRD peaks, as shown in figure 1(d), which can be assigned to (1 0 0), (1 1 0), and (2 0 0) diffractions of the 2-d hexagonal space group (*p*6 mm). As observed in the inset of figure 1(d), the two peaks at 2*θ* = 24.8° and 43.0° were (0 0 2) and (1 0 1) diffraction planes of hexagonal graphitic carbon. A broad peak around 24.8° indicates that the sample synthesized contains amorphous carbon. The diffraction peaks of N-OMC are stronger than those of OMC, which proves to intensify the graphitic degree of N-OMC [25].

The nitrogen adsorption–desorption isothermal was also employed to examine the surface area of N-OMC. As seen in figure 1(e) and table 1, a high specific surface area of 1062 m^2^ g^−1^ with an average pore diameter of 4.2 nm and specific pore volume of 0.98 cm^3^ g^−1^ can be observed.

**Table 1.** Surface area and pore structure parameters of N-OMC.

Sample	BET surface area (m^2^ g^−1^)	Average pore diameter (nm)	Pore volume (cm^3^ g^−1^)
N–OMC	1062	4.2	0.98

**Figure 2:**
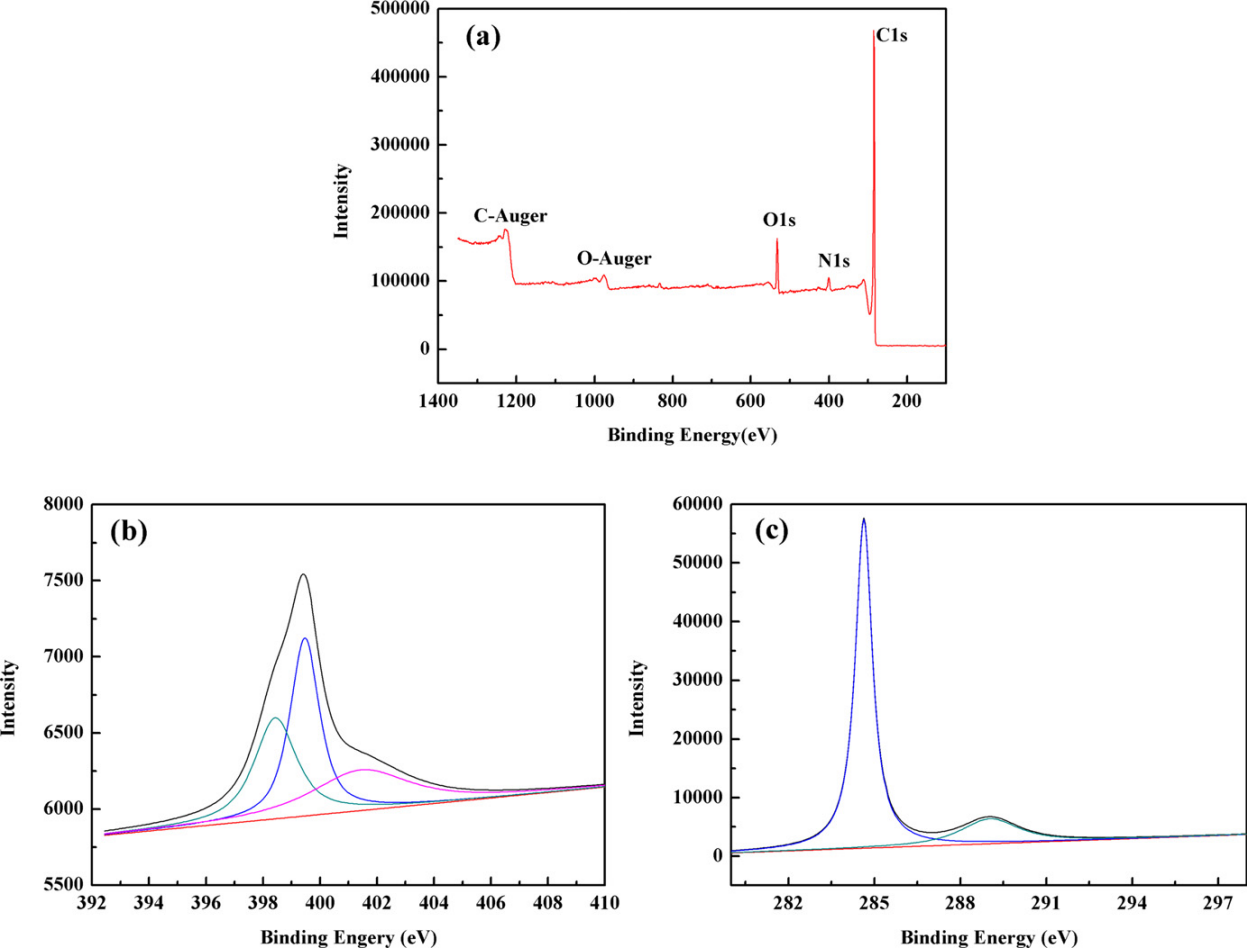
XPS spectra of the N-OMC (a), N1s (b) and C1s (c) of N-OMC.

The XPS spectra in figure 2(a) of N-OMC show strong signals from C, O and N elements. The surface nitrogen content measured from the XPS spectra was 3.42 wt%. The N1s spectrum in figure 2(b) can be well fitted to three components. The predominant component at 399.5 eV was assigned to N atoms trigonally bonded to three sp^2^ carbon atoms from the C-N network, indicating the formation of carbon nitride [26]. The peak at 398.5 eV can be assigned to the pyridinic-type nitrogen. A comparatively weak signal at 401.3 eV can be associated with several possible structures [26]. The C1s spectrum in figure 2(c) was deconvoluted into two peaks centered at 284.6 eV and 289.1 eV, respectively. The strongest signal at 284.6 eV was due to carbon-carbon bonding in a pure carbon environment of graphitic or amorphous carbon [27]. Furthermore, the peaks centered at 289.1 eV can be mainly assigned to the carbon atoms in quinine and/or pyridine and carboxyl groups [28].

**Figure 3:**
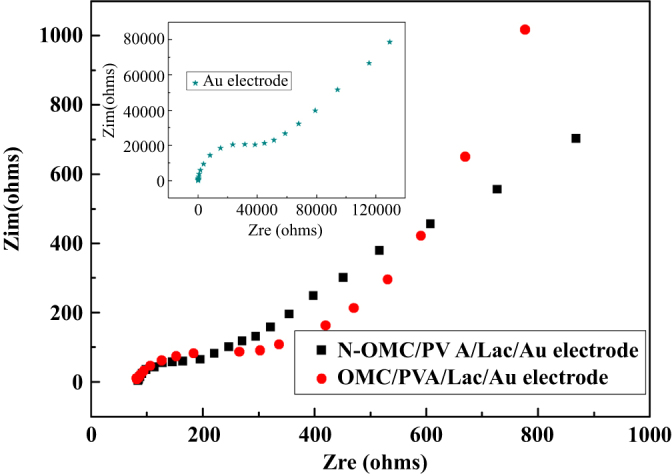
Nyquist plots of N-OMC/PVA/Lac/Au, OMC/PVA/Lac/Au and bare Au electrode (inset) in 5 mM K_3_[Fe(CN)_6_]/K_4_[Fe(CN)_6_]/0.1 M KCl solution.

EIS was used to investigate the electrochemical properties of N-OMC modified electrode surfaces [29]. By using a K_3_[Fe(CN)_6_]/K_4_[Fe(CN)_6_] redox couple as an electrochemical probe, the Nyquist plots of a bare Au electrode, N-OMC/PVA/Lac and an OMC/PVA/Lac modified electrode in the frequency range from 10^−2^ Hz to 10^5^ Hz were obtained in figure 3. It was observed that for the above-mentioned electrodes, the impedance spectra followed the theoretical shapes, a squeezed semicircle observed at high frequency, which corresponded to the electron transfer limited process, followed by a linear part at the low frequency attributable to diffusion controlled electron transfer process. In the low frequency region, no vertical increase in impedance on the imaginary part with decreasing the AC frequency was observed, which demonstrates that the electrodes exhibit no capacitive characteristics.

The respective semicircle diameters at the high frequency equal the electron transfer resistance (R_ct_) at the electrode surface. It was found that R_ct_ of the N-OMC/PVA/Lac modified Au electrode was about 180 *Ω*, which was smaller than 300 *Ω* of the OMC/PVA/Lac modified Au electrode and 4500 *Ω* of the bare Au electrode. The results indicated that N-OMC/PVA could act as a superior electron-transfer interface between the EIS probe and the electrode by accelerating the electron transfer rate on the electrode surface effectively. In addition, the smaller R_ct_ implied that the incorporation of N improved the electron transfer rate on electrodes surface effectively.

### Voltammetric behavior of catechol at the N-OMC/PVA/Lac/Au electrode

**Figure 4:**
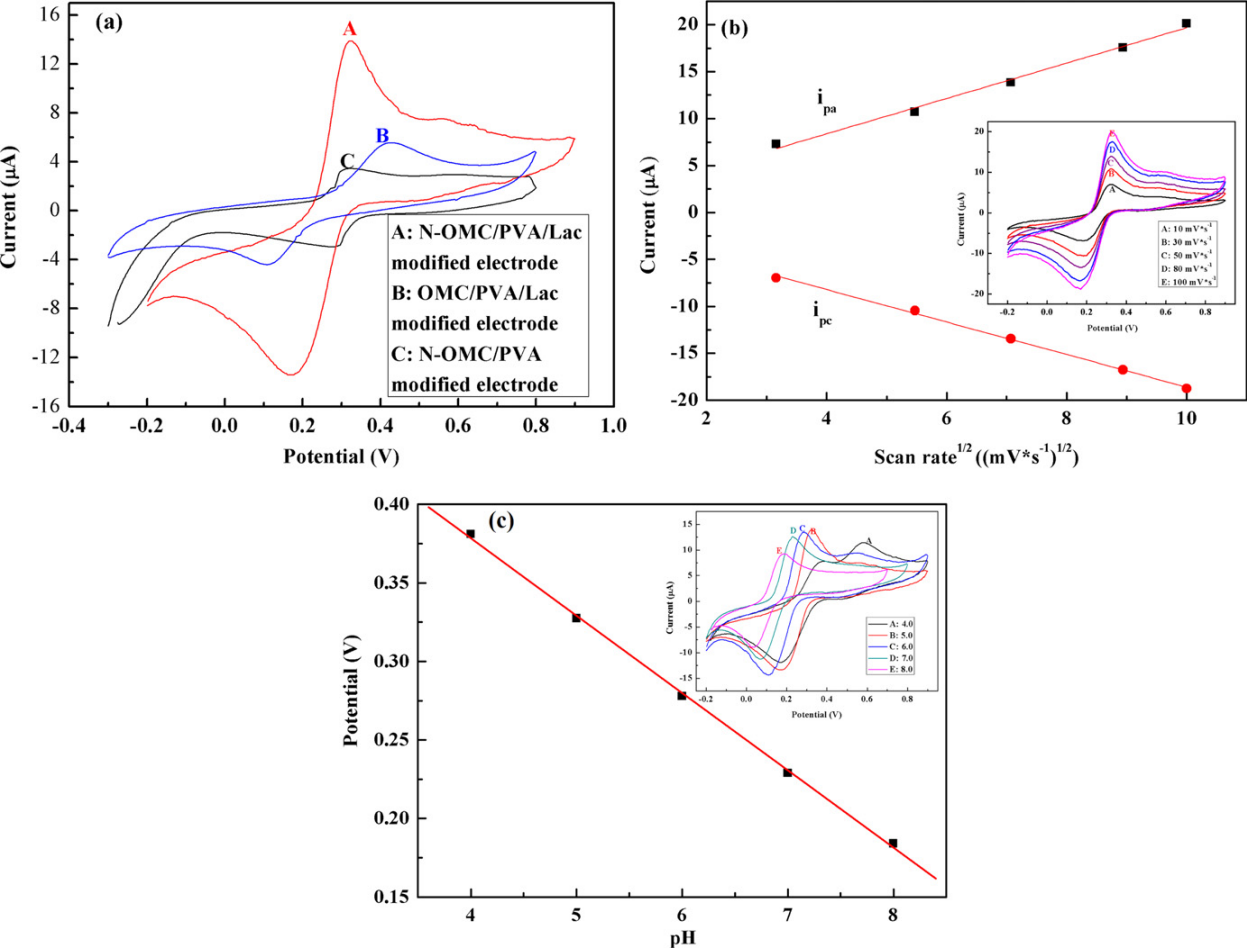
(a) CV plots of N-OMC/PVA/Lac/Au (A), OMC/PVA/Lac/Au (B) and N-OMC/PVA/Au (C) electrodes in 0.1 M PBS (pH 5.0) containing 0.05 mM catechol. (b) Current *versus* the square root of scan rate plots of N-OMC/PVA/Lac/Au electrode in 0.1 M PBS (pH 5.0) containing 0.05 mM catechol. Inset of figure 4(b): CV plots of the N-OMC/PVA/Lac/Au electrode in 0.1 M PBS (pH 5.0) containing 0.05 mM catechol at different scan rates from inner to outer: 10, 30, 50, 80, and 100 mV*s^−1^. (c) Calibration plot of pH *vs* potential. Inset of figure 4(c): CV plots of the N-OMC/PVA/Lac/Au electrode in 0.1 M PBS (pH 5.0) containing 0.05 mM catechol (pH: 4, 5, 6, 7 and 8).

Cyclic voltammograms (CVs) were used to investigate the catalytic activities of the N-OMC/PVA/Lac/Au electrode. Figure 4(a) shows CV plots of N-OMC/PVA/Lac/Au (A), OMC/PVA/Lac/Au (B) and N-OMC/PVA/Au (C) electrodes in 0.1 M PBS containing 0.05 mM catechol. As observed in figure 4(a), N-OMC/PVA/Au electrodes exhibit one pair of redox peaks ascribed to the oxidation of catechol, which proves the electrocatalytic properties of N-OMC towards catechol. The N-OMC/PVA/Lac/Au electrode also exhibits a pair of redox peaks with the anodic and cathodic peak potential positioned at +0.33 V and +0.18 V, which can be ascribed to the oxidation of catechol. However, the anodic peak potential of OMC/PVA/Lac/Au electrode shifted to a more positive value and the cathodic peak potential shifted in a lower negative direction. Moreover, the peak current at the N-OMC/PVA/Lac/Au electrode is 5 times that of N-OMC/PVA modified Au electrodes and 2.54 times that of the OMC/PVA/Lac/Au electrode. The lower oxidation potential and higher current response clearly indicates that N-OMC has excellent electrocatalytic activity towards catechol, which may be attributed to the co-oxidation of catechol by the laccase immobilized in N-OMC and N-OMC or the activating effect to laccase by N incorporated in N-OMC.

Figure 4(b) shows CV plots of catechol at the N-OMC/PVA/Lac/Au electrode at various scan rates. The anodic and cathodic peak currents increased linearly with the square root of the scan rate with a correlation coefficient of 0.997 and 0.998 (linear regression equations: i_pa_ = 1.88 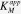 ^1/2^ + 0.86; i_pc_ = −1.73 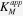 ^1/2^ − 1.26), which indicated a diffusion controlled process occurring on the surface of the N-OMC/PVA/Lac/Au electrode [30].

In the redox process of the electroactive protein on the electrode, there are usually protons involved. If protons participate in the process of the electrode, the peak potential values in the cyclic volt–ampere graphs of the oxidoreductase will shift along with pH changes of the electrolyte solution in the same direction. As shown in figure 4(c), the peak potential was closely dependent on pH of the solution, and the values of anodic peak potential shifted to more negative potentials with the increase of pH. In addition, it can be obviously observed that the anodic peak potential (Epa) with pH behaved in a well-defined linear relation with the slope of 57.5 mV*pH^−1^, which suggested that the overall process was proton dependent with an equal number of protons and electrons involved [31–33]. The separation of redox peaks in a 4.0 pH PBS was caused by the slower current induced by a larger proton gradient in the strong acid system.

### Optimization of the biosensor’s working conditions

**Figure 5:**
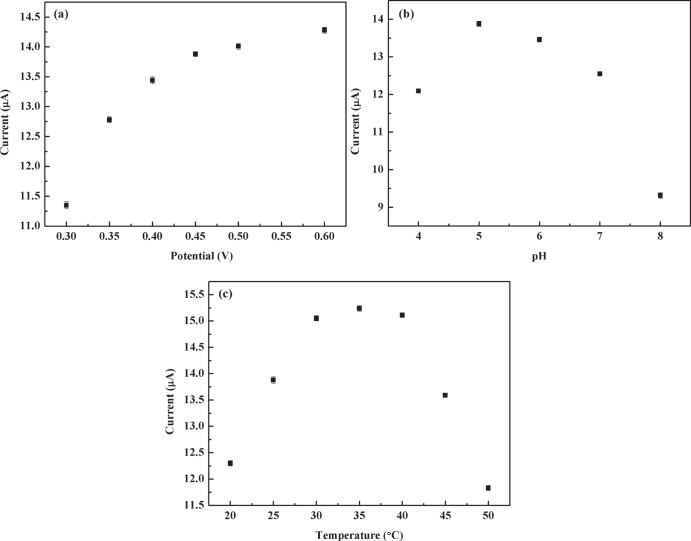
(a) Effect of applied potential on current response of the N-OMC/PVA/Lac/Au electrode to 0.05 mM catechol in 5.0 PBS. (b) Effect of pH of PBS on current response of the N-OMC/PVA/Lac/Au electrode to 0.05 mM catechol at 0.45 V (*vs* SCE). pH of PBS: 4.0, 5.0, 6.0, 7.0, 8.0. (c) Effect of temperature on current response of the N-OMC/PVA/Lac/Au electrode to 0.05 mM catechol in 5.0 PBS at 0.45 V (*vs* SCE).

The effects of applied potential, solution pH and temperature on the response current of the N-OMC/PVA/Lac/Au electrode to catechol were investigated in detail. From figure 5(a), the response current increased rapidly when the applied potential changed from 0.3 V to 0.45 V. But when the potential was higher than 0.45 V, the response current began to level off. In addition, the interference from the electroactive materials of pyrogallol and 2-amino phenol coexisting in the solution was relatively low at the low applied potential [34]. Thus, a potential of 0.45 V was selected as the working potential.

The effect of solution pH on response current was studied between pH 4.0 and 8.0. As shown in figure 5(b), the maximum response current was obtained at a pH of 5.0, which was similar to the *T*. *hirsuta* Lac modified graphite electrode [35, 36]. Therefore, it can be concluded that the Lac immobilized in the N-OMC/PVA composite matrix has higher bioactivity at about pH 5.0.

The effect of temperature on response current was presented in figure 5(c). The response current increased with the temperature from 20 to 35 °C, which can result from the increase of the enzyme reaction rate. But the response current began to decrease when the temperature was over 35 °C because of the denaturation of laccase. The response current reached the maximum at 35 °C. Thus, the temperature of 35 °C was chosen as the optimum temperature for the laccase biosensor.

### Current-time response of Lac immobilized on N-OMC/PVA

**Figure 6:**
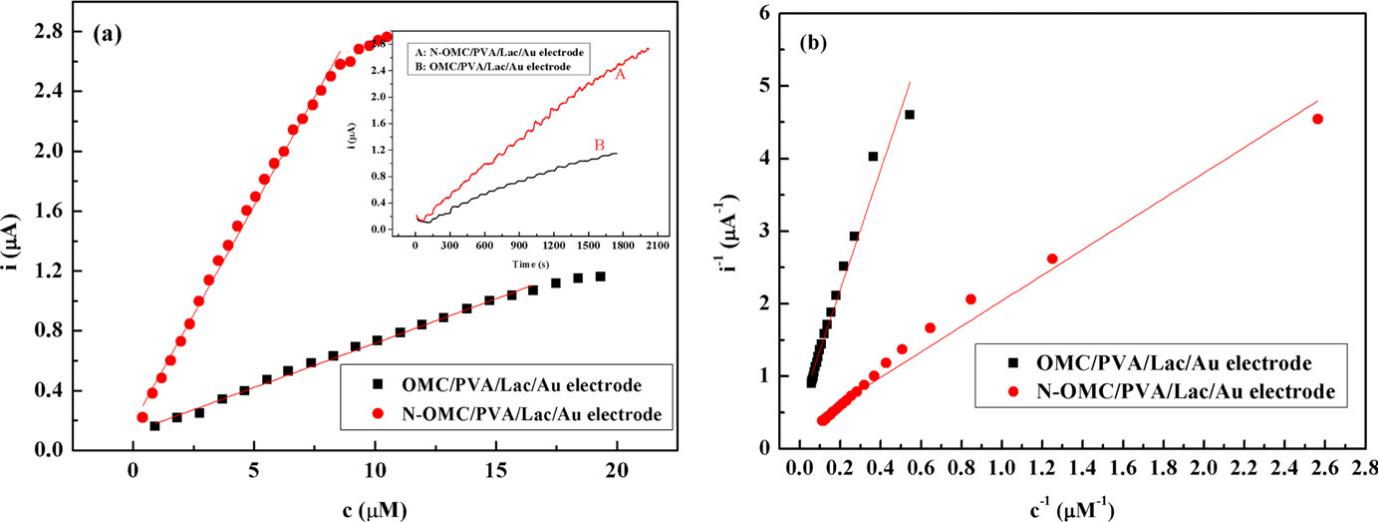
(a) The calibration curves of the N-OMC/PVA/Lac/Au electrode (A) and OMC/PVA/Lac/Au electrode (B) at 0.45 V (*vs* SCE). Inset: Amperometric responses of the N-OMC/PVA/Lac/Au electrode (A) and OMC/PVA/Lac/Au electrode (B) on successive additions obtained upon the successive addition of 0.5 mM catechol to stirred blank pH 5.0 PBS at 0.45 V (*vs* SCE). (b) The Lineweaver-Burk curves of the N-OMC/PVA/Lac/Au electrode (A) and OMC/PVA/Lac/Au electrode (B) at 0.45 V (*vs* SCE).

Figure 6(a) shows calibration curves of N-OMC/PVA/Lac/Au and OMC/PVA/Lac/Au electrodes to catechol. It can be seen that the response current increased with the increase of catechol concentration at low catechol concentration, and at high concentration, the current increased slowly and tended to become stable when the concentration of catechol was high enough, which indicated the characteristic of Michaelis–Menten kinetics. As observed in figure 6(a), the linear ranges of N-OMC/PVA/Lac/Au and OMC/PVA/Lac/Au electrodes were from 0.39 to 8.98 *μ*M and 0.92 to 16.55 *μ*M, and the sensitivities were 0.29 A*M^−1^ and 0.059 A*M^−1^, respectively. As listed in table 2, the detection limit of the N-OMC/PVA/Lac/Au electrode was 0.31 *μ*M, which was lower than 0.67 *μ*M of the Cu-OMC/PVA/Lac/Au electrode [21] and 0.331 *μ*M of the MB-MCM-41/PVA/Lac electrode [7].

**Table 2.** Analytical parameters obtained at different laccase biosensors.

Electrode material	Sensitivity (AM^−1^)	Linear range (*μ*M)	Detection limit (*μ*M)	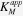 (*μ*M)
MB-MCM-41/PVA/Lac [7]	not applicable	4.0–87.98	0.331	256
Cu-OMC/PVA/Lac/Au [21]	0.104	0.67–15.75	0.67	40.2
carbon ceramic/Lac[37]	not applicable	0.1–10	0.06	123
carbon-fiber/Lac [38]	0.33 × 10^−3^	1–10	not applicable	610
Lac [38]	not applicable	not applicable	not applicable	3900
This work	0.29	0.39–8.98	0.31	6.28

Kinetic studies of the immobilized laccase were performed at various concentrations of catechol. The apparent Michaelis–Menten constant 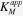) was calculated from the calibration plot using the Lineweaver–Burk plot (1/i versus 1/concentration). From the Lineweave–Burk curves in figure 6(b), the 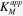 can be calculated to be 6.28 *μ*M (*R* = 0.9972, *n* = 23) for the N-OMC/PVA/Lac/Au electrode, and 15.3 *μ*M (*R* = 0.9962, *n* = 19) for OMC/PVA/Lac/Au, respectively. The 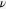 of 6.28 *μ*M was lower than 0.61 mM and 3.9 mM of 123 mM of the Lac-modified carbon ceramic electrode [37], fungal Lac immobilized on carbon-fiber electrodes [38], 0.256 mM of MB-MCM-41/PVA/Lac biosensor [7], and 40.2 *μ*M of the Cu-OMC/Lac/Au biosensor [21]. The smaller 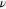 value indicated that the immobilized enzyme has higher enzymatic activity and implied that N-OMC will enhance the electrocatalysis of the substrate on the surface of electrodes.

### Repeatability, reproducibility and stability of the Lac biosensor

The reproducibility of the N-OMC/PVA/Lac/Au electrode was evaluated by comparing the response currents of 10 prepared enzyme electrodes. The relative standard deviation (RSD) was 6.2% when 0.5 mM catechol was determined. A reproducible current response with an RSD of 3.4% was observed for 30 successive assays of 0.5 mM catechol. The long-term stability was investigated by measuring the catechol solution intermittently, and the electrode was stored at 4 °C by immersing in PBS (0.1 M, pH 5.0) when it was not in use. The results showed that the response current maintained more than 95% of its initial value after 30 days, which indicated good stability.

### Analytical application

Some wine samples supplied by Grace Vineyard were selected for catechol determination using the proposed biosensor and the standard addition method. The results of the analysis using the biosensor were compared with those obtained using the standard spectrophotometric method in table 3. A t-test (95% confidence level) performed on these data showed that there was no significant difference between the results obtained with the two methods.

**Table 3.** Determination of catechol (mg l^−l^) in wine from Grace Vineyard using a standard method and the proposed biosensor.

Sample	Spectrophotometric method	Biosensor
A	10.41 ± 0.12	10.62 ± 0.1
B	10.11 ± 0.10	11.08 ± 0.12
C	9.87 ± 0.10	9.97 ± 0.12

## Conclusions

In the present study, a functionalized N-OMC with good electrocatalytic properties has been synthesized. With the prominent advantages of large surface area, uniform mesopores and remarkable electrocatalytic properties, an amperometric biosensor was developed by immobilizing Lac into the pores of N-OMC. The enzyme molecules assembled on N-OMC/PVA exhibited high bioactivity and stability, and N-OMC was demonstrated as suitable candidates to immobilize enzymes. The assembled laccase biosensor exhibited high sensitivity, low detection limit, good stability and acceptable reproducibility for the determination of catechol. This work demonstrates that the N-OMC/PVA composite provides a support for laccase immobilization and construction of biosensors.
